# The axis IL-10/claudin-10 is implicated in the modulation of aggressiveness of melanoma cells by B-1 lymphocytes

**DOI:** 10.1371/journal.pone.0187333

**Published:** 2017-11-16

**Authors:** Elizabeth Cristina Perez, Patricia Xander, Maria Fernanda Lucatelli Laurindo, Ronni Rômulo Novaes e Brito, Bruno Camolese Vivanco, Renato Arruda Mortara, Mario Mariano, José Daniel Lopes, Alexandre Castro Keller

**Affiliations:** 1 Environmental and Experimental Pathology Program, Universidade Paulista, São Paulo, São Paulo, Brazil; 2 Department of Microbiology, Immunology and Parasitology, Universidade Federal de São Paulo—Escola Paulista de Medicina (UNIFESP/EPM), São Paulo, São Paulo, Brazil; 3 Department of Pharmaceutics Sciences, Universidade Federal de São Paulo, Campus Diadema, Diadema São Paulo, Brazil; 4 Centro Universitário São Camilo, São Paulo, São Paulo, Brazil; 5 Department of Medicine, Nephrology Division, Universidade Federal de São Paulo–Escola Paulista de Medicina (UNIFESP/EPM), São Paulo, São Paulo, Brazil; IDI, Istituto Dermopatico dell'Immacolata, ITALY

## Abstract

B-1 lymphocytes are known to increase the metastatic potential of B16F10 melanoma cells via the extracellular signal-regulated kinase (ERK) pathway. Since IL-10 is associated with B-1 cells performance, we hypothesized that IL-10 could be implicated in the progression of melanoma. In the present work, we found that the C57BL/6 mice, inoculated with B16F10 cells that were co-cultivated with B-1 lymphocytes from IL-10 knockout mice, developed fewer metastatic nodules than the ones which were injected with the melanoma cells that were cultivated in the presence of wild-type B-1 cells. The impairment of metastatic potential of the B16F10 cells was correlated with low activation of the ERK signaling pathway, supporting the idea that the production of IL-10 by B-1 cells influences the behavior of the tumor. A microarray analysis of the B-1 lymphocytes revealed that IL-10 deficiency is associated with down-regulation of the genes that code for claudin-10, a protein that is involved in cell-to-cell contact and that has been linked to lung adenocarcinoma. In order to determine the impact of claudin-10 in the cross-talk between B-1 lymphocytes and the B16F10 tumor cells, we took advantage of small interfering RNA. The silencing of claudin-10 gene in B-1 lymphocytes inhibited activation of the ERK pathway and abrogated the B-1-induced aggressive behavior of the B16F10 cells. Thus, our findings suggest that the axis IL-10/claudin-10 is a promising target for the development of therapeutic agents against aggressive melanoma.

## Introduction

B-1 lymphocytes are part of the innate immune system and have distinct roles in inflammation, infection, and resistance to tumors. They constitute the central B-cell population in the mice peritoneal and pleural cavities but rarely occur in spleen and lymph nodes [1, 2]. These cells contribute toward the maintenance of natural IgM levels and are the major source of IL-10, which is a regulatory cytokine involved in the downregulation of immune responses. In response to the activation of the innate immunity, B-1 lymphocytes tend to increase both natural IgM and IL-10 levels, which are very important for the development of resistance to pathogens and also the modulation of several immune-mediated inflammatory responses [3, 4]. Despite an ever-growing interest in the role of B-1 cells in various immunological responses, their participation in resistance or susceptibility to tumors has been neglected. Thus, the aim of this study was to determine the involvement of B-1 cells in the behavior of B16F10 melanoma cells.

Using a heterotypic co-culture system, we demonstrated that upon contact with B-1 lymphocytes, the B16F10 melanoma cells increased the activation of the ERK signaling pathway and up-regulated the expression of MMP-9, CXCR4, and MUC18, which in turn, led to increased tumor growth and metastatic spreading [5–7]. We also showed that the functional and phenotypic alterations that are observed in melanoma cells post contact with B-1 lymphocytes were dependent on the IL-10 production by the latter [6]. As B-1 lymphocytes produce and utilize IL-10 as an autocrine growth factor [4], we hypothesized that this cytokine plays a significant role in the interplay between B-1 and B16F10 cells.

In the current work, the differential gene expression between B-1 lymphocytes isolated from the wild-type (B-1WT) or the IL-10 knockout (B-1IL-10^-/-^) mice was determined by the microarray analysis. Among seven genes that displayed different expression patterns, claudin-10, a component of tight junction strands, was chosen for functional analysis because it has been reported to be associated with lung adenocarcinoma [8]. To this end, we used small interfering RNA (siRNA) to silence the claudin-10 expression in B-1 lymphocytes and evaluated the effects on the metabolism of the B16F10 cells. The silencing of claudin-10 expression in B-1 lymphocytes impairs the ability of the cells to induce the ERK phosphorylation in the B16F10 cells and to increase the aggressiveness of the tumor. Thus, our results demonstrate that the axis IL-10/claudin-10 is a suitable target to control the behavior of melanoma cells.

## Materials and methods

### Mice

Female wild-type (WT), B cell-deficient (BKO) and IL-10 knockout (IL-10-/-) C57BL/6 mice, 6–8 weeks old, were obtained from Centro de Desenvolvimento de Modelos Experimentais para Medicina e Biologia (CEDEME), Brazil. All mice were housed in a pathogen-free facility and fed with autoclaved water and solid standard chow. This study was carried out in strict accordance with the recommendations in the Guide for the Care and Use of Laboratory Animals of the National Institutes of Health. This study was approved by the Ethics Committee of the Universidade Federal de São Paulo, Brazil (Permit Number: 0799/08). All euthanasia was performed using cervical dislocation method and all efforts were made to minimize suffering.

### Cell culture

B16F10 melanoma cells were cultured in RPMI-1640 (Sigma, St Louis, MO, USA) containing 10% of fetal bovine serum (Cultilab, Campinas SP, Brazil) antibiotics and supplements. B-1 lymphocytes enriched fraction from wild-type (B-1WT) or IL-10^-/-^ (B-1IL-10^-/-^) C57BL/6 mice were obtained by culture of five days of total peritoneal cells. As previously described [6], free-floating cells correspond mostly to B-1 lymphocytes. The total peritoneal cells were stained with anti-CD19, and anti-CD23 antibodies (BD Biosciences, USA) and the B-1 lymphocytes were separated by electronic cell sorting of CD19+CD23- cells (FACS Aria II cell sorter, BD Biosciences, USA).

### Purification of B-1 lymphocytes

Purified B-1 lymphocytes ex vivo were obtained from the peritoneal cavity of WT mice by successive washed with RPMI medium. The cell suspension was labeled with anti-mouse CD19 APC and anti-mouse CD23 FITC (both from BD Biosciences, Mountain View, CA, USA) and then submitted to electronic cell sorting using a FACSAria II cell sorter (BD Biosciences). Finally, single B-1 lymphocytes (CD19+CD23-, pB-1WT) and other cells (non-B-1WT) were collected separately.

### Microarray sample preparation, Gene-chip hybridization and analysis

Trizol reagent (Invitrogen Corporation, Carlsbad, CA, USA) was used to isolate total RNA from enriched cultures of B-1 WT or IL-10-/- lymphocytes. Three biological replicates of each sample were used for the array hybridizations. The sample preparation, hybridization, and microarray data analysis on GeneChip® mouse genome 430 2.0 array (Affymetrix, Santa Clara, CA, USA) were performed and analyzed as described [9]. Briefly, images were first examined for visible defects using GeneChip ® Operating Software (GCOS). Signal intensities for each gene and data normalization were performed using RMA method (Robust Multi-Array Analysis) [10] with RMA express software [11]. Differentially expressed genes were identified using SAM (Significance Analysis of Microarray) with MultiExperiment Viewer (MeV), software deployed by The Institute of Genomic Research–TIGR, Rockville, MD. The criteria for selecting differentially expressed genes between B-1 lymphocytes from WT and IL-10^-/-^ mice was performed using fold change ≥2 and false selection rate (FDR–false discovery rate) ≤4%. The microarray data are available at the ArrayExpress under protocol accession E-MEXP-3973.

### Transfection with siRNA

Three Stealth RNAi™ siRNA sequences were synthesized commercially by Invitrogen Life Technologies Inc. (USA) with the help of tools available online (http://www.invitrogen.com). Stealth RNAiTM siRNA1 (RNA)-AUCAUUAGUCCUCUACAUGCCUGGA (cldn-10 siRNA1, primer number 148163E05), Stealth RNAiTM siRNA2 (RNA)-UCCAGGCAUGUAGAGGACUAAUGAU (cldn-10 siRNA2, primer number 148163E06), Stealth RNAiTM siRNA3 (RNA)-UUUGCAUACAGGGAACAGCCUGUCA (cldn-10 siRNA3, primer number 148163E07) were designed to target claudin-10 mRNA. The basic local alignment search tool (BLAST) was carried out to verify the target of the three sequences. All sequences have the ability to suppress any of six claudin-10 isoforms[12]. Stealth RNAi™ siRNA Negative Control (negative siRNA 12935–300, Ambion®/Life Technologies Corporation, CA, USA) was used to check nonspecific cellular events caused by the introduction of the oligonucleotide into cells. For transfection, B16F10 cells or B-1 lymphocytes enriched culture were collected and seeded at 0.5x10^6^ cells per well in a 12-well plate. Then, cells were transfected or not with 100 nM of claudin-10 siRNAi1, siRNAi2 or siRNAi3 or with negative control siRNA, according to the manufacturer’s protocol for siPORT™ NeoFX™ Transfection Agent (InvitrogenTM, Life Technology Inc. CA, USA). The transfection efficiency of each Stealth RNAi™ siRNA sequence was confirmed by flow cytometry using Silencer® Cy™3 Labeled GAPDH (AM 4623, Ambion®/ Life Technologies Inc. CA, USA). The western blot analyses confirmed the silencing of claudin-10 expression.

### Co-cultures of B16F10 cells and B-1 lymphocytes

As previously described 1x10^5^ B16F10 cells were co-cultured with 1x10^6^ B-1 lymphocytes in 6-well plate [6]. After 48 hours without changing the medium, non-adherent cells (B-1 lymphocytes) were removed, and adherent cells (B16F10 cells) were washed for three times with phosphate-buffered saline (PBS) to remove all the remaining B-1 lymphocytes. B16F10 cells were collected using trypsin (Cultilab, Brazil) for subsequent assays. To determine the purity of B16F10 cells after co-cultures, they were incubated with CD11b and IgM antibodies (BD Biosciences, USA) for flow cytometry analysis. The data acquisition was performed in a FACSCalibur using CELLQUEST software (BD Biosciences, USA), and data were analyzed using FlowJo® software (Tree Star Inc., USA).

### Experimental metastasis assays

B16F10 melanoma cells recovered from culture were injected into the lateral tail vein of syngeneic C57BL/6 mice (1x10^5^cells/animal). After fourteen days, mice were euthanized, and the number of lung tumor colonies was determined.

### Total cellular protein extracts and western blot

Total protein extraction was performed as previously described [6]. Anti-phospho-ERK (E-4, sc-7383), total ERK (K-23, sc-94) from Santa Cruz Biotechnology (Santa Cruz, CA, USA), anti-claudin-10 (38–8400) from Invitrogen (Invitrogen Corporation, CA), anti-β actin (Sigma, St Louis, MO) or anti-GAPDH (AB9485) both from Abcam® (Abcam®, Cambridge, USA) were used as primary antibodies. Peroxidase-conjugated goat anti-mouse IgG (Sigma, St Louis, MO) and anti-rabbit (BioRad, Hercules, CA, USA) were used as secondary antibodies. The reactions were developed using a chemiluminescence ECL kit (Amersham Pharmacia, Uppsala, Sweden). Bands were visualized using UVItec (UVItec Limited, Cambridge, UK) and analyses were performed using Uviband 1D analysis software (UVItec Limited, Cambridge, UK).

### Statistical analysis

All data represent at least three independent experiments, expressed as the mean ± the standard deviation. Statistical comparisons were made by ANOVA followed by Turkey Multiple Comparison Test. Analyses were performed with GraphPad Prism 4.0 software (GraphPad Software Inc., San Diego, CA, USA). Values of p<0.05 were considered statistically significant.

## Results

### B-1 lymphocytes, but not the other peritoneal cell population, increase the metastatic behavior of melanoma cells in an IL-10-dependent manner

**Fig 1:**
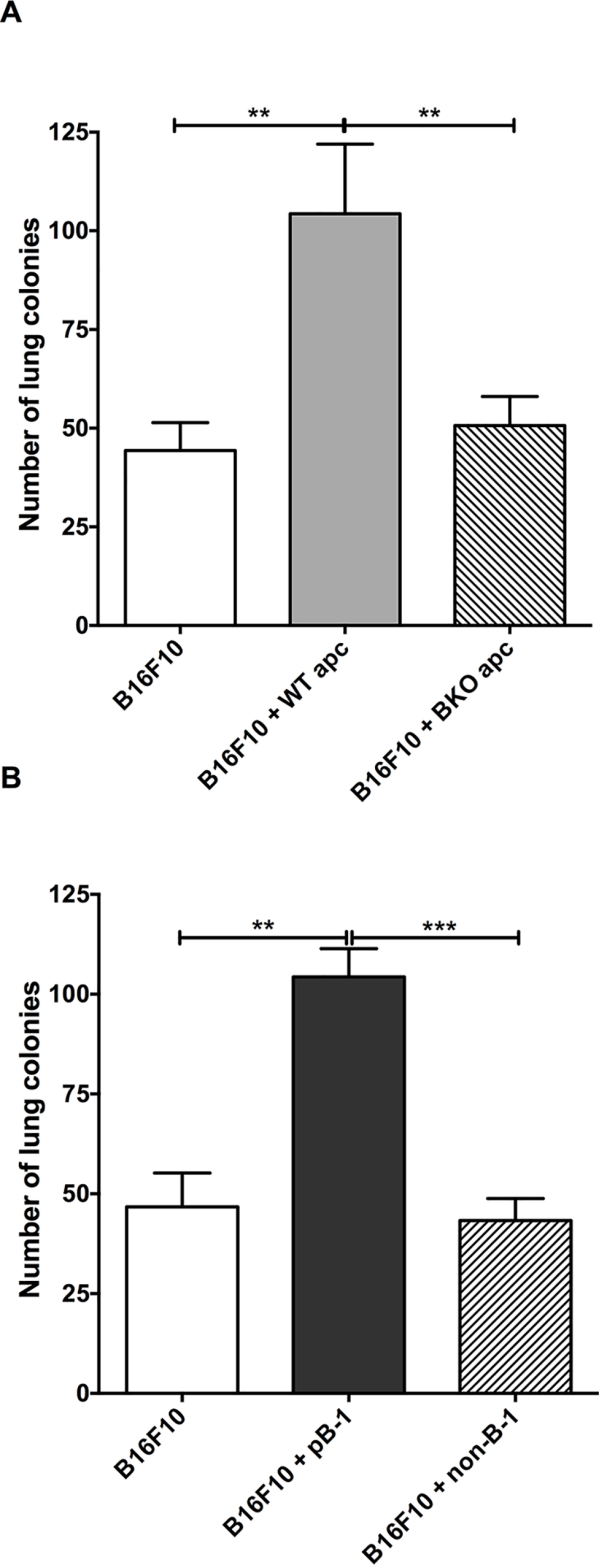
B-1 lymphocytes, but not other peritoneal cells, induce the pro-metastatic effect on B16F10 melanoma cells. **A)** Adherent peritoneal cells (apc) from wild-type mice, but not from B cell-deficient mice (BKO), obtained from enriched peritoneal cell cultures increases B16F10 metastatic potential upon cell-to-cell contact. **B)** The same results were obtained with cell-sorting technique, confirming that B-1 lymphocytes are responsible for changes in the melanoma behavior. (Bars represent the mean number of lung tumor per experimental condition ±SD (*n* = 4); **p < 0.01; ***p < 0.001, using one-way ANOVA with Tukey’s post hoc test.

To prove the non-engagement of the peritoneal cells other than the B-1 lymphocytes in the pro-metastatic effect on the B16F10 melanoma cells, we took advantage of the B cell-deficient (BKO) C57/BL6 mice. The BKO mice are deficient in both the B-1 and the B-2 lymphocytes. Briefly, total peritoneal cells were isolated from the wild-type (WT) or the BKO mice and were cultured for 40 min followed by the removal of non-adherent cells. The WT adherent peritoneal cells (WTapc) were collected and co-cultured with the B16F10 melanoma cells at 10:1 ratio for 48 h. The mice which received B16F10 co-cultivated with WTapc exhibited more metastatic nodules than the ones which were injected with B16F10 cells, either cultivated alone or with the BKO mice adherent peritoneal cells (BKOapc, Fig 1A). To further confirm that B-1 lymphocytes are responsible for the aggressiveness of the tumor, B16F10 melanoma cells were co-cultivated with purified peritoneal B-1 lymphocytes (CD19^+^CD23^-^; S1 Fig) or the non-B-1 peritoneal cells. As depicted in the Fig 1B, only the purified B-1 lymphocytes (B16+pB-1) enhanced the metastatic potential of the B16F10 melanoma cells.

### Endogenous IL-10 drives the pro-metastatic effect of B-1 lymphocytes on B16F10 melanoma cells

**Fig 2:**
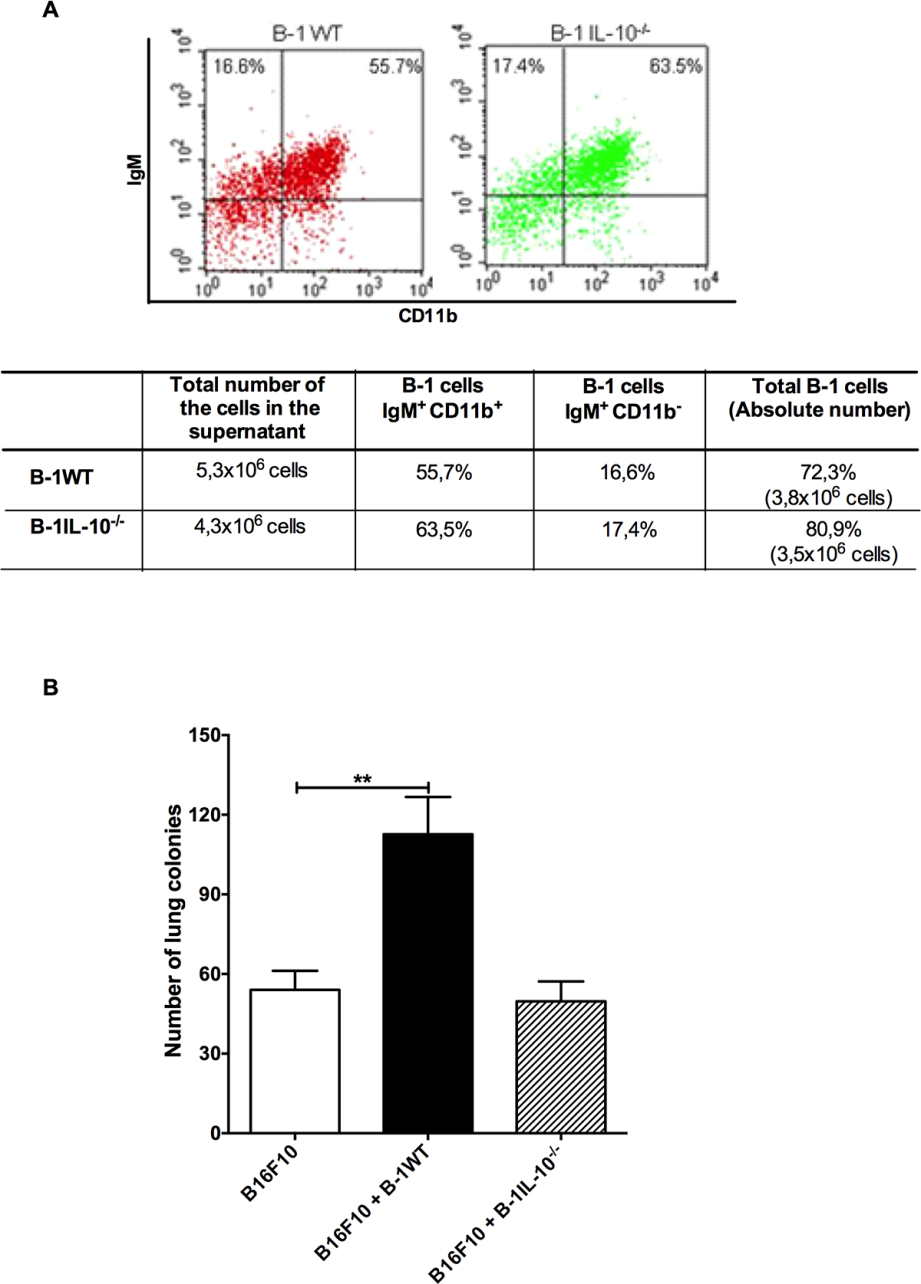
Endogenous IL-10 drives the influence of B-1 lymphocytes in B16F10 behavior. **A)** The deficiency in IL-10 production did not affect the phenotype and the number of B-1 lymphocytes recovered from the culture of peritoneal cavity cells; **B)** but affected their ability to increase the metastatic potential of B16F10 melanoma cells. Bars represent the mean number of lung colonies per experimental condition ±SD. **p <0.001, using one-way ANOVA with Tukey’s post hoc test.

Although earlier studies have reported that the addition of exogenous IL-10 to melanoma cultures has no effect on the B16F10 behavior [6], we hypothesized that endogenous IL-10 could influence the metabolism of B-1 lymphocytes; thereby indirectly affecting the B16F10 metastatic potential. To test this, we checked by flow cytometry analysis phenotypic differences between B-1 lymphocytes from WT and IL-10^-/-^ mice. Fig 2A illustrates that there was no significant difference, neither in the phenotype nor in the total number of cells that were recovered after the culture of peritoneal cells from the WT or the IL-10^-/-^ mice. In contrast, the animals that were inoculated with the B16F10 cells co-cultured with B-1WT lymphocytes presented a higher number of lung melanoma colonies in comparison to the mice that received B16F10 cells cultivated alone or co-cultured with the B-1IL-10^-/-^ lymphocytes (Fig 2B). Consequently, such data supported the notion that the influence of B-1 lymphocytes in the behavior of melanoma cells depends upon the endogenous IL-10 levels.

### IL-10 deficiency impairs the expression of claudin-10 in B-1 lymphocytes

**Fig 3:**
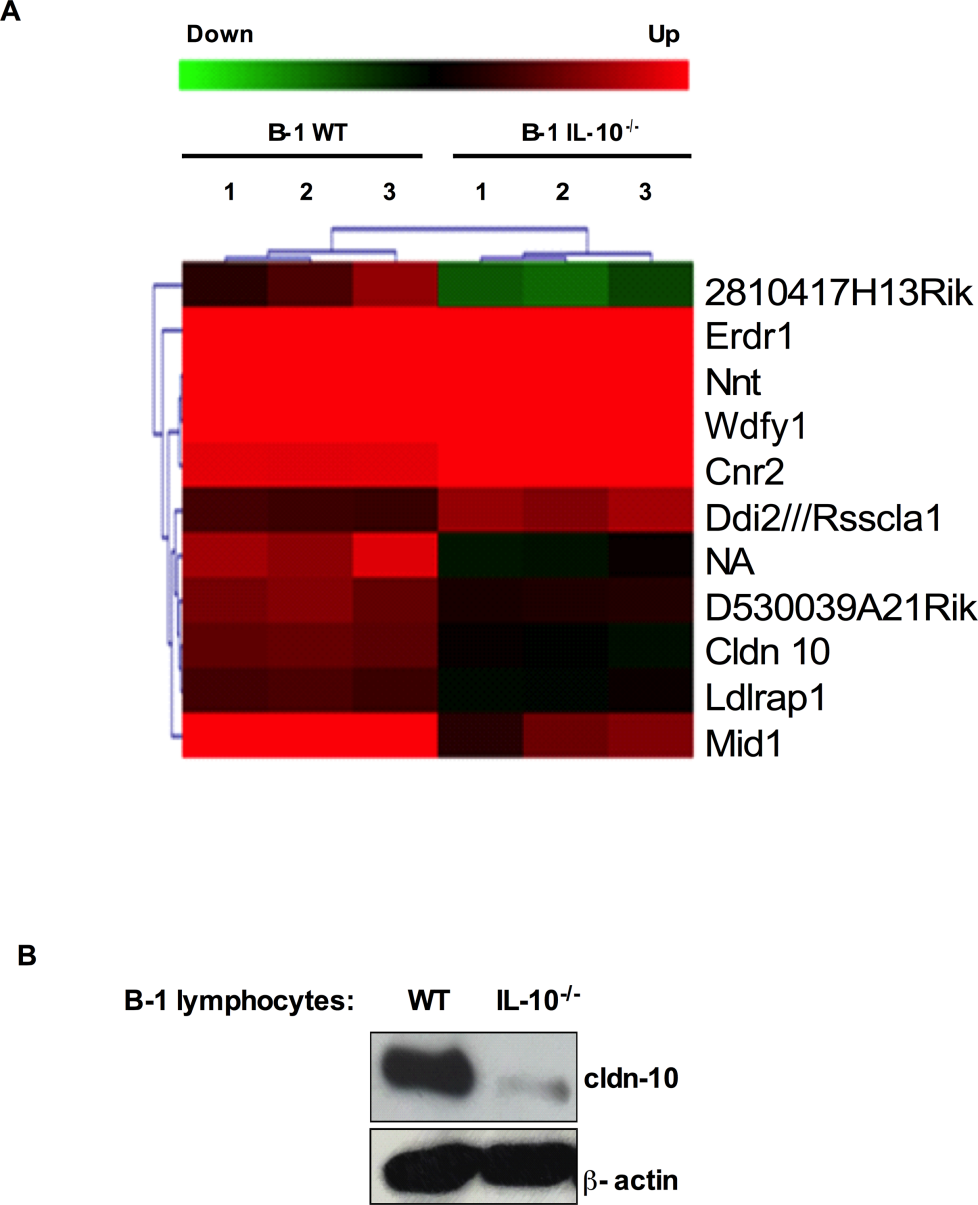
IL-10 deficiency is associated with impaired expression of claudin-10 in B-1 lymphocytes. Total RNA of B-1 lymphocytes enriched culture from wild-type (B-1WT) or IL-10^-/-^ knockout (B-1IL-10^-/-^) mice was submitted to microarray analysis. **A)** Heatmap showing differential expression of eleven transcripts between B-1WT and B-1IL-10^-/-^ lymphocytes, and the down-regulation of claudin-10 (cldn-10) in B-1IL-10^-/-^ samples. **B)** Western blot analysis of cldn-10 protein in whole-cell extracts from B-1WT and B-1IL-10^-/-^. β-actin protein was used as internal control.

Since the flow cytometry analysis did not reveal any phenotypic differences between the B-1WT and the B-1IL-10^-/-^ cells (Fig 2A), we used the high throughput microarray technique to obtain the gene expression profiles of these cells. The comparison analyses were done using the MEV software, and the statistically significant transcripts were selected by the unpaired two-class SAM analysis (FDR and Q-value 4%) by applying 500 random permutations. Using these parameters, the chip analysis revealed the variations in the expression of eleven (11) genes between the WT and the IL10^-/-^ B-1 lymphocytes (Fig 3A). The analysis of the regulated gene clusters by the DAVID Functional Annotation Chart tool displayed relevant differences between the WT and the IL-10^-/-^ B-1 lymphocytes in a group of five genes: three up-regulated (cldn-10, mid-1, ldlrap1) and two down-regulated (cnr2 and nnt). Among these five (5) genes, we validated the expression of claudin-10 (cldn-10) transcript because the claudin family of proteins has been associated, either positively or negatively, with the progression of various tumors [13–16]. In accordance with the microarray results, the western blot analysis also showed the down-regulation of claudin-10 in the B-1IL-10^-/-^ lymphocytes as compared to the B-1WT cells (Fig 3B).

### Claudin-10 mediates the interplay between B-1 lymphocytes and melanoma cells

**Fig 4:**
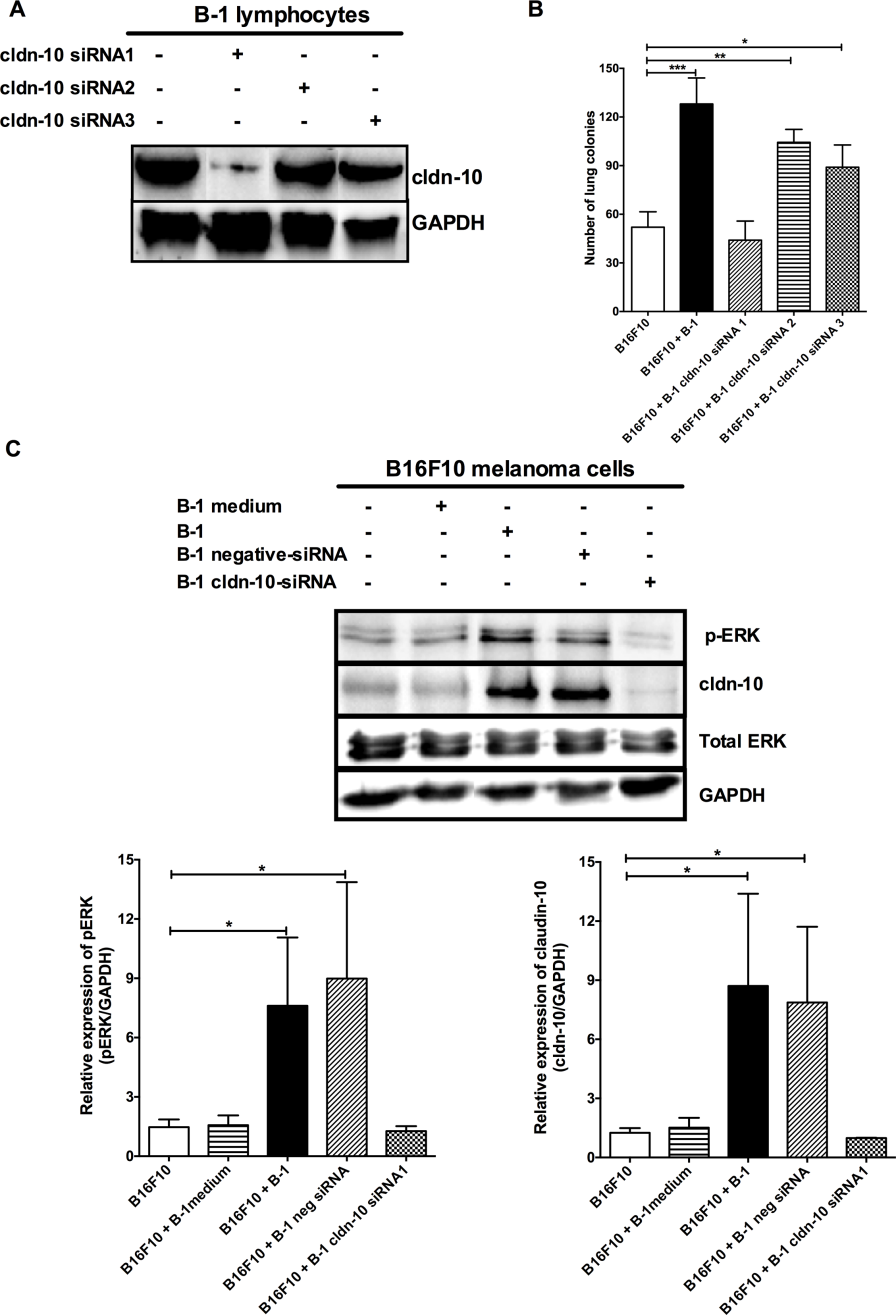
Claudin-10 expression on B-1 lymphocytes mediates the alterations in B16F10 melanoma cell behavior. **A)** Expression of claudin-10 on B-1 lymphocytes from peritoneal cell culture transfected for 24 h with stealth claudin-10 siRNA1 (B-1 cldn-10 siRNA1), siRNA2 (B-1 cldn-10 siRNA2), siRNA3 (B-1 cldn-10 siRNA3). **B)** The number of lung colonies in mice injected with B16F10 melanoma cells from single culture (B16F10) or after co-cultivation with B-1 lymphocytes (B16F10+B1), transfected or not with claudin-10 siRNA1 (B16F10 + B-1 cldn-10siRNA1), siRNA2 (B16F10 + B-1 cldn-10 siRNA2), siRNA3 (B16F10 + B-1 cldn-10 siRNA3). **C)** Inhibition of claudin-10 expression impairs the activation of ERK pathway and the claudin-10 expression in B16F10 cells after B-1 contact. GAPDH was used as internal control. Bars represent the mean number of lung colonies per experimental condition ±SD. *p < 0.05; **p < 0.01;***p < 0.001, using one-way ANOVA with Tukey’s post hoc test.

In an attempt to verify the role of claudin-10 gene in our model, we used the Stealth RNAi^TM^ siRNA technology. The B-1WT lymphocytes were transfected with claudin-10 siRNA1, siRNA2 or siRNA3 sequences and subjected to western blotting to verify the abrogation of transcription of the claudin-10 gene. Among the three siRNAs, only the siRNA1 exhibited significant inhibition of the claudin-10 gene expression in B-1 cells (Fig 4A). To determine the role of claudin-10 in the pro-metastatic effect of B-1 lymphocytes, the B16F10 cells were co-cultured for 48 h with B-1 lymphocytes, untransfected or transfected, with claudin-10 siRNAs followed by experimental metastasis assay. The inhibition of claudin-10 expression by siRNA1 impaired the B-1 lymphocyte mediated increase of the metastatic potential of B16F10 cells (Fig 4B). In contrast, the mice, which were inoculated with the B16F10 cells co-cultured with the siRNA2 or siRNA3 transfected or untransfected B1 cells, demonstrated the highest number of lung melanoma metastases (Fig 4B). In addition, the suppression of the claudin-10 expression in B-1 lymphocytes inhibited the ERK pathway activation in B16F10 cells post contact with B-1 lymphocytes (Fig 4C). Similarly, increase in claudin-10 expression in the B16F10 cells upon contact with B-1 lymphocytes was also inhibited, indicating that the ERK pathway is involved in the expression of claudin-10 in the melanoma cells. Of note, B-1 lymphocytes that were co-transfected with the negative control siRNA displayed the expression of claudin-10 gene and the pro-metastatic influence on the B16F10 cells (S2 Fig). This finding signified that only the claudin-10 gene silencing specifically led to the observed phenomenon.

**Fig 5:**
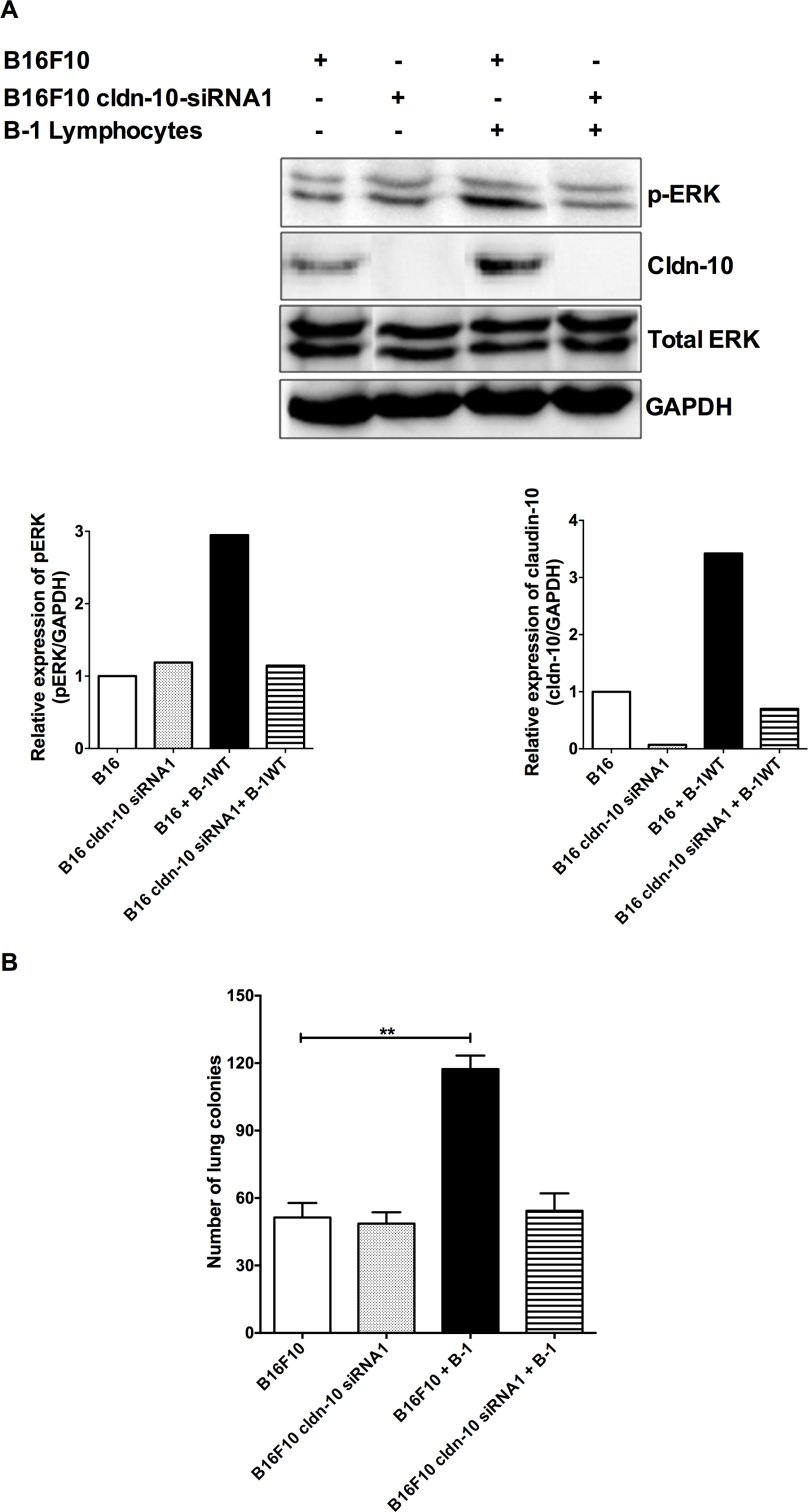
Inhibition of claudin-10 expression in B16F10 inhibits the influence of B-1 lymphocytes in melanoma aggressiveness. B16F10 cells were transfected for 24h with stealth claudin-10 siRNA1. The inhibition of claudin-10 expression in B16F10 blocks **A)** activation of ERK pathway and claudin-10 expression and, **B)** impairs the B16F10 aggressiveness induced by the contact with B-1 lymphocytes. (Bars represent the mean number of lung tumor per experimental condition ±SD (*n* = 3). **p < 0.01; ***p < 0.001, using one-way ANOVA with Tukey’s post hoc test.

Finally, to further confirm the role of claudin-10 in bringing out the changes that are observed in the melanoma cells after contact with B-1 lymphocytes, the claudin-10 expression was inhibited in the B16F10 cells. As hypothesized, in the absence of claudin-10, B-1 lymphocytes were unable to modulate the metastatic behavior of the B16F10 melanoma cells (Fig 5). Thus, our findings demonstrate that claudin-10 is a key mediator in the interplay between B-1 lymphocytes and B16F10 melanoma cells and is involved in the enhancement of melanoma aggressiveness upon contact with the B-1 cells.

## Discussion

Like the other cells of the immune system, B-1 lymphocytes can be associated with protective or deleterious responses depending on the context in which they are activated [3, 4, 17–19]. Concerning the involvement of B-1 lymphocytes in tumor behavior, we found that analogous to tumor-associated macrophages (TAMs), the B-1 lymphocytes seem to promote growth and spread of melanoma cells [5, 6, 20, 21]. Previously, we reported that the pro-metastatic effect of B-1 lymphocytes on the B16F10 melanoma cells was dependent upon the cell-to-cell contact and the ERK signaling, however, the mechanisms involved in this phenomenon remained unclear [5, 6, 9]. Since IL-10 is known to play an important role in B-1 biology, we hypothesized that the cytokine could be involved in the behavior alteration that is observed in the case of the B16F10 melanoma cells.

In the present study, we demonstrated that the peritoneal cells other than the B-1 lymphocytes were not able to boost the melanoma aggressiveness. Although a previous study demonstrated that IL-10, itself, was unable to modulate the B16F10 behavior, its influence on the cross-talk between the B-1 lymphocytes and the melanoma cells was not clear [6]. Thus, we co-cultured the B16F10 cells with B-1 lymphocytes that were isolated from IL-10^-/-^ mice. The IL-10-deficient B-1 lymphocytes were unable to induce alterations in the metastatic melanoma behavior, indicating that the IL-10 production by B-1 lymphocytes plays a pivotal role in the interplay between these cells.

Furthermore, in an attempt to investigate the influence of IL-10 in B-1 cell biology, we evaluated the effect of IL-10 in gene expression profile of the B-1 lymphocytes. The microarray analysis revealed differential expression of eleven (11) transcripts among the B-1WT and B-1IL-10^-/-^ lymphocytes, which was in concordance with the hypothesis that the endogenous IL-10 plays crucial role in the B-1 physiology. Among the 11 transcripts, claudin-10 was downregulated in the IL-10^-/-^ B-1 lymphocytes and seemed to be the most important mediator in the BF16/B-1 interaction. Firstly, the claudin family of proteins presents the major integral proteins that constitute the backbone of tight junctions, being involved in cell-to-cell contact [22] and; secondly, the claudin gene expression may be regulated by the ERK signaling pathway [6, 23], which is up-regulated in both the B16F10 cells and the B-1 lymphocytes after contact with each other [6, 23].

To determine the influence of claudin-10 in the interaction between the B-1-lymphocytes and the melanoma cells, claudin-10 expression was inhibited using RNAi approach. The suppression of claudin-10 levels in the B-1 lymphocytes impaired the ability of the latter to increase the aggressiveness of melanoma cells, which was accomplished by the down-modulation of the ERK signaling pathway (Fig 4C). Thus data from the present work corroborated the previous findings that established an association between the ERK signaling and the tumor aggressiveness [6] and, supporting the notion that claudin-10 is involved in the changes in melanoma cells upon contact with B-1 lymphocytes. In addition, similar results were recorded when claudin-10 was inhibited in the B16F10 cells, validating the role of claudin-10 as a mediator to increase the metastatic potential of B16F10 melanoma after contact with B-1 lymphocytes. Together, these data support the hypothesis that homotypic interactions mediated by claudin-10 are required to trigger the more metastatic behavior on B16F10 melanoma cells after contact with B-1 lymphocytes.

## Conclusions

In summary, our findings demonstrate that endogenous IL-10 influences the expression of claudin-10 in B-1 lymphocytes and implicate the axis IL-10/claudin-10 to augment the aggressive behavior of the B16F10 melanoma cells upon contact with B-1 lymphocytes. Thus, the identification of claudin-10 as a mediator of the pro-metastatic effect of B-1 lymphocytes on melanoma cells reveals a potential target for the development of molecular therapeutics to control the melanoma metastasis.

## Supporting information

S1 Fig Gate strategy for B-1 lymphocyte purification by flow cytometry after cell culture. The gate strategy resulted in single cells with purity of > 99.5% after electronic cell sorting.
(TIFF)

S2 Fig The siRNA methodology has no effect on B1-lymphocyte ability to promote B16F10 aggressiveness. B-1 lymphocytes were transfected for 24 h with claudin-10 siRNA or negative siRNA to determine the impact of stealth methodology in their activity on B16F10 cells. A) The inhibition of claudin-10 expression depends on the specific siRNA used. B) The negative stealth siRNAi has no effect on B-1 lymphocytes' ability to promote further the metastatic behavior in B16F10 melanoma cells. Data are the mean ± SD of three independent experiments. ***p < 0.0001, using one-way ANOVA with Tukey’s post hoc test.
(TIFF)
