# Supplementary figures and images for: The axis IL-10/claudin-10 is implicated in the modulation of aggressiveness of melanoma cells by B-1 lymphocytes
Source: PLoS One. 2017 Nov 16;12(11):e0187333. doi: 10.1371/journal.pone.0187333 (PMC5690663; doi:10.1371/journal.pone.0187333)

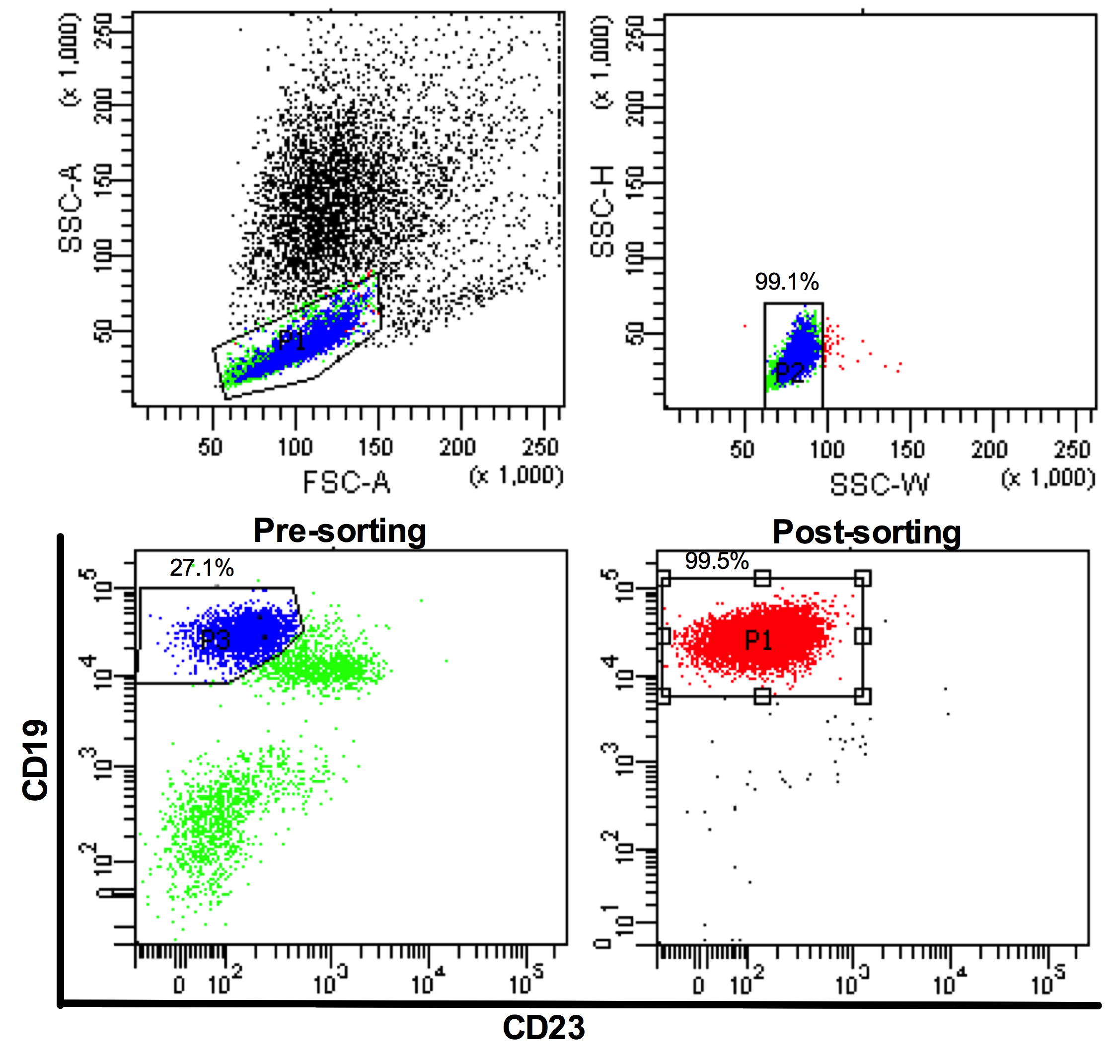

Supplement: S1 Fig — The gate strategy resulted in single cells with purity of > 99.5% after electronic cell sorting. (TIFF) [file pone.0187333.s001.tiff]

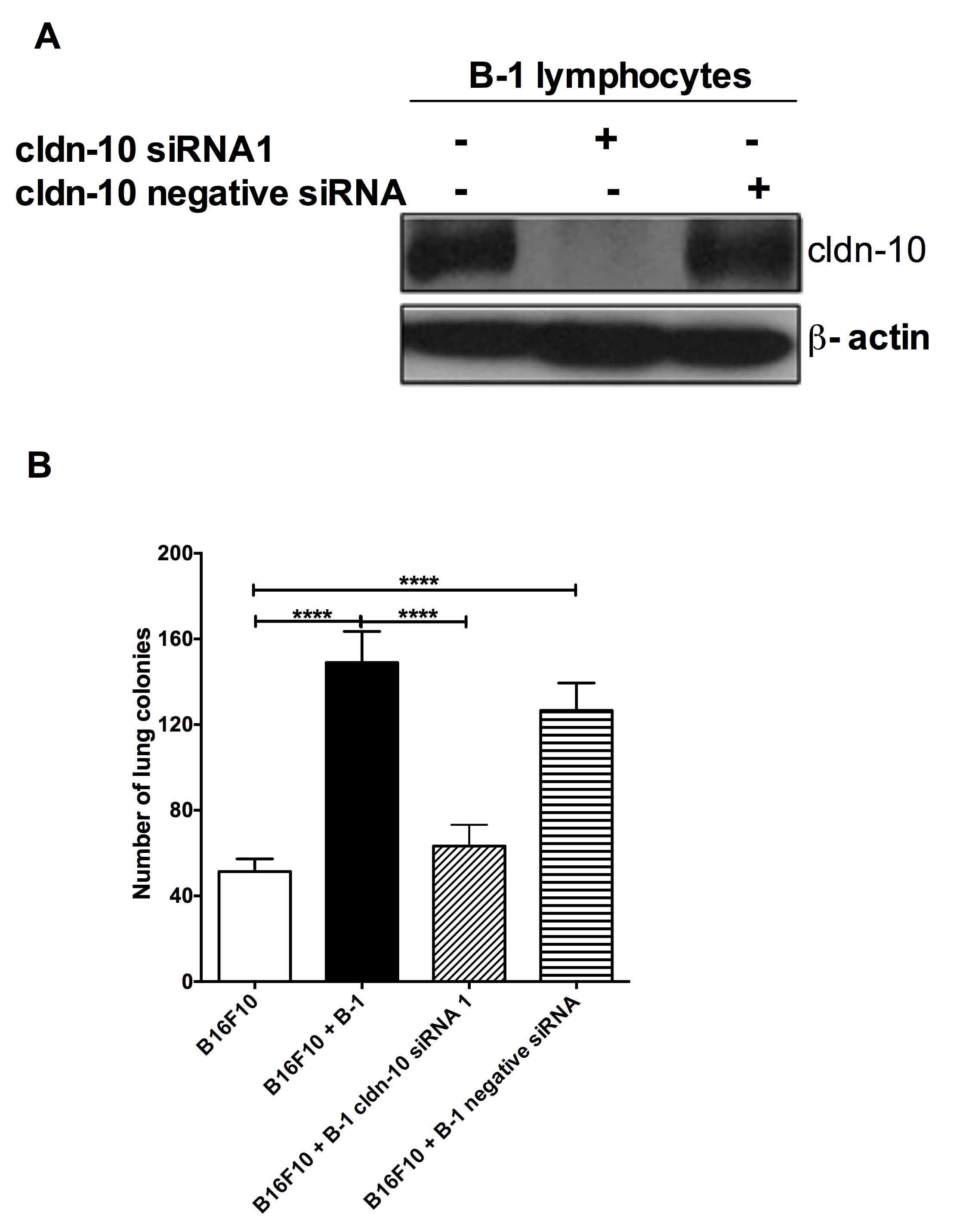

Supplement: S2 Fig — B-1 lymphocytes were transfected for 24 h with claudin-10 siRNA or negative siRNA to determine the impact of stealth methodology in their activity on B16F10 cells. A) The inhibition of claudin-10 expression depends on the specific siRNA used. B) The negative stealth siRNAi has no effect on B-1 lymphocytes' ability to promote further the metastatic behavior in B16F10 melanoma cells. Data are the mean ± SD of three independent experiments. ***p < 0.0001, using one-way ANOVA with Tukey’s post hoc test. (TIFF) [file pone.0187333.s002.tiff]
